# Weaving Invisible Threads: The Impact of Caregiver Trauma on Parenting and Child Brain Development

**DOI:** 10.3390/brainsci15070726

**Published:** 2025-07-07

**Authors:** Manessa Riser, Christopher Trentacosta, Tanja Jovanovic

**Affiliations:** 1 Department of Psychiatry and Behavioral Neurosciences, Wayne State School of Medicine, Detroit, MI 48201, USA; ck0524@wayne.edu; 2 Department of Psychology, Wayne State University, Detroit, MI 48202, USA; chris.trentacosta@wayne.edu

**Keywords:** trauma exposure, parenting, brain development, intergenerational trauma

## Abstract

Trauma exposure has severe consequences for the exposed individuals as well as their children. The current paper is a conceptual narrative review that uses the intergenerational transmission of trauma (ITT) framework to synthesize the literature on the association between a parent’s exposure to trauma and the brain and behavioral outcomes in their children. There are likely multiple mechanisms for the ways in which parental history of trauma exposure can lead to these negative outcomes. However, this review highlights parenting as a key pathway through which caregiver trauma conveys both the risk and resilience in the child’s neurodevelopment, emphasizing the need for trauma-informed, relationship-focused interventions.

## 1. Introduction

Parenting plays a central role in child developmental outcomes, serving both as a protective factor and a pathway for risk, especially in the context of trauma exposure. Trauma can include events involving actual or threatened death, serious injury, or sexual violence, as well as several forms of childhood maltreatment (physical, sexual, or emotional) that are typically assessed as occurring prior to age 18. Childhood trauma is also commonly characterized by experiences that threaten the physical or emotional safety and overwhelm a child’s capacity to cope, such as abuse, neglect, or witnessing violence. Even further, rates of trauma exposure are disproportionately high in low-income and urban populations [1]. A growing body of literature suggests that when caregivers have a personal history of trauma (hereafter referred to as “parental trauma”), it can profoundly shape how they parent, leading to disruptions in emotional attunement, attachment security, and regulation strategies that are essential to healthy parent–child relationships. These disruptions in caregiving can, in turn, affect not only children’s behavioral outcomes but also their neurobiological development. For example, findings from a 2020 study by Wu et al. suggest that children exposed to lower-quality parenting demonstrated a higher prevalence of developmental delays compared to those receiving higher-quality parenting, based on multiple follow-up visits between the ages of 1 and 4 years [2]. Despite the mounting evidence supporting these associations, there is limited integrative work that bridges the psychological, behavioral, and neurodevelopmental pathways through which parental trauma influences offspring brain development. This paper addresses that gap by applying the intergenerational transmission of trauma (ITT) framework for understanding how caregiver trauma impacts parenting and how that in turn affects children. ITT centers the relational and behavioral pathways that contribute to the cycle of trauma, explicitly framing parenting as mechanisms of risk and resilience [3]. We expand upon Lang and Gartstein’s ITT framework that focused on the perinatal period by reviewing links between parental trauma exposure and outcomes across infancy, childhood, and adolescence with an emphasis on neurobiological processes and outcomes.

Rather than conducting a systematic review, this paper adopts a conceptual approach informed by the ITT framework that synthesizes key empirical studies to demonstrate how trauma-related disruptions in parenting may influence the development of child brain systems, particularly systems responsible for stress regulation, emotion processing, and executive functioning. The literature was selected on its conceptual and theoretical relevance to the overarching ITT framework with a focus on studies that examined links between caregiver trauma, parenting behaviors, and child developmental outcomes. Relevant empirical papers were identified by the first author (MR) through targeted searches in academic databases (e.g., PubMed, Google Scholar, etc.). Search terms including combinations of keywords such as “parental trauma”, “caregiver PTSD”, “parenting behaviors”, “child brain development”, “intergenerational trauma”, “childhood maltreatment”, and “neurodevelopment” were used to ensure coverage, but inclusion was determined by the degree to which each study contributed meaningfully to the conceptual synthesis. Preference was given to peer-reviewed empirical studies published within the past two decades, though seminal older studies were included when foundational to the field. While this is not a systematic review, efforts were made to ensure that selected studies were conceptually and methodologically aligned with the framework, with attention to diversity in study designs, populations, and outcome measures.

This paper presents a conceptual, theory-driven review of how parenting behaviors contribute to the intergenerational transmission of trauma (ITT). Using the ITT framework, we synthesize the evidence linking caregiver trauma histories, parenting behaviors, and child outcomes. Our review aims to identify key mechanisms, particularly relating to parenting, which may mediate or moderate the transmission of trauma across generations, and to propose directions for future research and intervention. The paper is organized around three major components. First, we examine the influence of parental trauma on parenting behaviors, with attention to specific mechanisms such as emotion dysregulation, attachment disruptions, and parenting style. Second, we explore many of the downstream consequences of these disruptions for children’s psychological and neurobiological outcomes, including changes in brain structure, connectivity, and stress physiology. Finally, we provide a brief review of emerging interventions that target either the parent, the parent–child relationship, or family-level processes to mitigate intergenerational risk and promote resilience. Here we also targeted interventions that might mitigate neurobiological outcomes in the child.

Our goal is to highlight studies that demonstrate how trauma-exposed parenting impacts the development of prefrontal-limbic circuits, stress response systems (e.g., hypothalamic–pituitary–adrenal (HPA) axis dysregulation), and behavioral patterns such as anxiety, aggression, or emotion dysregulation in children. In doing so, we aim to clarify how specific parenting dimensions mediate or moderate the effects of trauma and to identify points of intervention that may foster adaptive neurodevelopmental outcomes.

## 2. Parental Factors

### 2.1. Trauma Exposure in Parents

Globally, up to 90% of individuals will experience at least one traumatic event during their lifetime [4]. As a result of trauma exposure, some may develop post-traumatic stress disorder (PTSD) with a reported lifetime prevalence of 5.6% [5]. Millions of individuals affected by trauma are also navigating the challenges of parenthood, which is a period of extensive neurobiological changes resulting from the social and psychological changes related to caregiving [6]. The literature suggests that parental trauma exposure and trauma-related psychopathologies, like PTSD, contribute to adverse parenting behaviors [7] and subsequent negative child developmental outcomes [8]. When a parent experiences trauma—whether through direct exposure to violent events like war and combat, or different forms of childhood maltreatment like physical or sexual abuse—they often struggle to engage in healthy, adaptive parenting behaviors. Parents with a history of trauma exposure exhibit poorer overall parenting behaviors [9,10,11,12] and more significant potential for abuse [10]. These maladaptive behaviors may lead to long-term consequences for the child’s psychological, emotional, and behavioral well-being.

Over the past two decades, research has focused on understanding how a parent’s history of trauma exposure shapes parenting behaviors, with particular emphasis on emotion dysregulation [13], attachment challenges [14,15,16], and maladaptive parenting styles [10]. Research has shown associations between parental trauma exposure and adverse developmental outcomes in children, including PTSD symptoms, internalizing problems like anxiety and depression, and externalizing behaviors like violence and defiance [8,11]. Persistent associations between parental trauma and child psychopathology, even without direct trauma exposure in the child, point to transgenerational pathways of risk transmission not solely explained by shared experiences [17]. Numerous studies have begun to reveal potential mechanisms through which trauma exposure in parents impacts offspring outcomes, providing insight into psychological, biological, and physiological consequences in the children of trauma survivors. By weaving together behavioral and neural data, this review proposes a conceptual model informed by the ITT framework in which parenting is a central channel through which the effects of trauma are biologically and behaviorally embedded in the next generation.

### 2.2. Trauma Exposure and Parenting Behaviors

The nuances of parenting are shaped by multiple factors, such as cultural context, socioeconomic status, and individual experiences, including the parent’s history of trauma. Parental trauma exposure can have long-term implications that influence a myriad of developmental outcomes in offspring, through biological and psychosocial mechanisms. In mothers with a history of childhood maltreatment, determined by the Structured Clinical Interview for DSM-IV (SCID), decreased cortisol has been linked to flatter affect during infant interactions [18]. This contrasts with findings from a prior study indicating that increased cortisol levels mediated the association between maternal childhood maltreatment, determined by the Childhood Trauma Questionnaire (CTQ) and greater parenting insensitivity [19]. Among a sample of 49 women aged 18–45, a history of childhood abuse was linked to elevated autonomic reactivity. Notably, the presence of current depressive symptoms significantly intensified this response by more than sixfold suggesting an interaction effect between early trauma and current mood on physiological stress sensitivity [20]. This autonomic dysfunction, in conjunction with abnormal amygdala-prefrontal connectivity, may create the perfect storm for heightened fear responses and reduced fear inhibition [21].

The impact of parental trauma may extend beyond biological mechanisms into psychosocial spheres of parenting. The caregiver–child relationship is shaped by a complex interplay of dimensions and styles, which can profoundly influence developmental outcomes in children [22]. Dimensions of parenting, such as warmth, responsiveness, control, and discipline, are foundational in determining the emotional and behavioral outcomes of offspring. These dimensions are expressed through various parenting styles, which are typically categorized as authoritative, authoritarian, permissive, or neglectful [23]. Authoritative parenting, marked by high warmth and moderate control, is often associated with positive child outcomes, including higher self-esteem, better emotional regulation, and engagement in school activities [24]. In contrast, authoritarian parenting, characterized by high control and low warmth, can lead to increased anxiety in adolescents [25]. Permissive and neglectful parenting, defined by low control with varying degrees of warmth, are linked to challenges such as poor self-discipline and emotional difficulties that may manifest as delinquency in youth between the ages of 10–19 [26]. These findings suggest that the impact of parenting on child and adolescent outcomes may vary depending on the developmental stage.

Trauma exposure has the potential to disrupt normative parenting. For example, parents who have experienced trauma often exhibit altered emotional responses and behavioral patterns that disrupt the nature of their care across multiple domains [10]. A study conducted in an urban sample of 176 mothers found that total cumulative trauma, measured by the Life Events Checklist of the Clinician-Administered PTSD Scale, significantly predicted dose-dependent increases across four parenting domains: physical discipline, punitiveness, psychological aggression, and potential for abuse [10]. Disrupted parenting can present as inconsistent, unsupportive, and cold. A caregiver’s exposure to trauma may impair their parenting by increasing negative emotionality, which is often associated with more permissive or lax parenting behaviors [15,27], increased irritability and aggression [28], emotion dysregulation [13], and overall dysfunctional parenting behavior [7] leading to negative consequences in offspring. Conversely, improvements in parents’ emotion regulation and stress management can foster more consistent and nurturing caregiving, thereby promoting positive outcomes in children [7]. Furthermore, it is important to recognize that specific dimensions of parenting have distinct effects on outcomes. Maternal warmth, specifically, has been linked to more positive outcomes. Stevens et al. found that maternal warmth attenuated amygdala reactivity in children [29] alluding to protective buffering of maladaptive neural activity. In conjunction with responsive parenting, maternal warmth has also been shown to be inversely associated with depressive and trauma-related symptoms in children during early childhood [30] and children transitioning between late childhood and early adolescence [31] who have experienced trauma. Conversely, higher levels of parental overcontrol may predict the trajectory of anxiety in children, leading to an increase in symptoms [32], while more harsh parenting is associated with higher rates of externalizing behaviors in children [33].

Moreover, an abundance of converging research suggests that parenting challenges are often influenced by parental psychopathology, specifically depression and PTSD. In a 2019 study with a sample of 185 mother-child dyads, Robinson and colleagues found that maternal trauma history was directly associated with anxiety symptoms in preschool-aged children, the quality of parenting, and maternal depressive symptoms [34]. Maternal depression [35] and maternal mental distress [36] have been shown to mediate the association between maternal trauma history and adverse outcomes in children of low-income families. Socioeconomic status may further compound this association, as low-income mothers often face higher rates of trauma exposure and depressive symptoms, which can intensify parenting challenges [37]. Research conducted by Muzik et al. (2017) echoes these findings, identifying maternal depression in mothers with a history of child abuse as a prominent factor contributing to parental impairment [38]. These associations may even be specific to the type of trauma exposure, as mothers with current experiences of interpersonal violence (IPV) were found to engage in more negative parenting practices, with childhood IPV exposure of varying types strengthening that association [39].

A personal history of traumatic experiences may cause parents to struggle with appropriately regulating their emotions, which subsequently disrupt healthy attachments with their offspring. Trauma exposure in early life has been linked to insecure attachment styles in parents, which may lead to disorganized or avoidant parenting patterns [14]. For example, parents with a history of childhood maltreatment may find it difficult to attend to their child, which could manifest as emotional unavailability or parental withdrawal [40]. This lack of emotional availability can undermine the child’s secure attachment development, resulting in behavioral and emotional difficulties later in life. Similarly, mothers with PTSD, compared to those without, exhibit more lax parenting behaviors, which are characterized by permissiveness and inconsistency with the association being mediated by higher levels of emotion dysregulation [41]. Results from a more recent study by Powers et al. [7] found that emotion dysregulation was positively correlated with parenting features such as over-reactivity and laxness, which aligns with findings from Kumar et al. [41] and negatively correlated with parental warmth in mothers.

Furthermore, trauma exposure can lead to heightened parenting stress [42], which is strongly correlated with maladaptive parenting behaviors. For instance, trauma-exposed parents are more likely to engage in harsh discipline or threatening parenting practices, such as verbal aggression or physical punishment [10,43,44]. These maladaptive parenting styles are not only harmful in the immediate context but can also contribute to the intergenerational transmission of trauma, as children may grow up to model these patterns in their own lives.

## 3. Child Developmental Outcomes

### 3.1. Parental Trauma and Child Psychological Outcomes

Children raised by adults with trauma exposure often experience significant psychological challenges, which are directly and indirectly associated with the behavioral manifestations of their parents’ symptoms [45]. Early maternal trauma exposure predicts overall negative effects [46], attentional biases to danger [47], anxiety [34], behavioral dysregulation [46], PTSD symptoms [48], and emotion dysregulation [49,50] in their children. A recent systematic review of twelve studies found significant intergenerational effects of maternal childhood maltreatment on child mental health problems [51]. In a study specific to female veterans, mothers’ PTSD symptomatology was related to an increase in offspring’s internalizing and externalizing symptoms [52]. Similarly, in non-military trauma cohorts, PTSD in mothers was associated with sleep-related anxiety in their infants [53]. Although most of the research has focused on mothers, similar trends are seen in studies of fathers with PTSD. For example, a longitudinal study found that paternal PTSD predicted poorer offspring mental health [54]. While parental trauma has been linked to various psychopathological features such as depression [28], the literature most often highlights its association with anxiety in children.

### 3.2. Parental Trauma and Child Brain Development

The children of parents with histories of trauma are at an elevated risk of experiencing biological and physiological alterations [55], which may contribute to difficulties regulating their emotions and behaviors. Dysregulation is marked by changes in the stress response systems, particularly within the HPA axis. Parents with PTSD can exhibit either heightened or blunted cortisol reactivity [56], which can alter the child’s stress physiology through genetic and environmental pathways. Altered cortisol levels have been observed in the offspring of parents who experienced trauma associated with the Holocaust [57], the terror attack on 9/11 [58], and other traumatic experiences during their childhood [59,60,61,62]. Similarly, blunted cortical reactivity was seen in the children of mothers with PTSD exposed to a laboratory stress paradigm [56]. Thus, the alteration of cortisol levels and reactivity may underlie the linked vulnerability between parent and child in relation to parental trauma.

Neurodevelopmental changes in the children of parents with PTSD also represent a critical area for concern. Structural, functional, and connectivity abnormalities in brain regions associated with emotion regulation, such as the prefrontal cortex, amygdala, and hippocampus have been reported in fetuses [63], newborns (range = 5 to 64 days post birth) [64], infants (range = 4–10 months) [40,64,65,66], and young children (mean age = 57.9 months) [67] of mothers who experienced childhood trauma and heightened stress during pregnancy. For instance, 319 infants were scanned using resting-state functional magnetic resonance imaging (rs-fMRI) to determine the association between neonatal brain connectivity and maternal experiences of neighborhood crime [66]. This study yielded two interesting findings. First, functional connectivity between the default mode network and the thalamus was weaker with higher levels of violent neighborhood crime. Secondly, higher levels of maternal exposure to violent crime predicted weaker functional connectivity between the amygdala and hippocampus. These findings provide insight into the impact of maternal trauma exposure on neural networks crucial for the development of frontal and limbic brain functional connectivity. Reduced connectivity between the prefrontal cortex and amygdala can impair the child’s capacity for emotion regulation [67,68] potentially leading to increased reactivity or maladaptive coping behaviors.

Furthermore, Moog et al. [64] found that maternal childhood maltreatment was associated with reduced intracranial volume in their newborns, 5–64 days old. Another study examined the impact of maternal childhood maltreatment on infant gray and white matter volume, specifically in the amygdala and hippocampal regions [40]. These regions are integral components of the fear neurocircuitry that are often impacted by trauma [69] and are of particular interest because of their role in emotion and memory processing [70,71]. Results demonstrated that maternal childhood maltreatment was associated with lower total brain volumes and gray matter volumes in their infants, between 4 and 24 months [72]. High maternal childhood maltreatment severity was also linked to lower right amygdalar volume as the age increased; however, there were no significant associations found with hippocampal volumes [72]. These neural alterations may be shaped by both direct genetic inheritance and the child’s exposure to parenting behaviors influenced by the parent’s traumatic history, such as overprotectiveness or excessive withdrawal. Similarly, an intergenerational trauma study including forty-five mother-child dyads linked higher emotion dysregulation in mothers to lower white matter connectivity in their older children (age 8–13 years) [73], specifically in the mean diffusivity of the fornix, which is a white matter structure critical for emotion and cognitive processing [74].

The offspring of parents with traumatic childhood histories also exhibit exaggerated startle responses, relating to hypervigilance, which is a hallmark symptom of PTSD. This may be a consequence of adverse changes in brain connectivity between cortical and limbic regions outlined above. Jovanovic and colleagues [75] found that children of mothers with elevated levels of abuse, specifically physical abuse, had increased dark-enhance startle (DES) responses. This association remained significant even when controlling for child’s trauma exposure and maternal symptoms of PTSD. A subsequent study in the same population revealed a significant association between maternal emotion dysregulation and elevated DES in her child, which was fully mediated by parenting stress [76]. Moreover, in a 2021 study of school-aged children (ages 8–13), Stenson and colleagues examined the physiological response to threat and safety learning [77]. The study included 137 dyads of mothers and children. Children completed a fear conditioning paradigm, in which they acquired fear of a danger signal (reinforced conditioned stimulus; CS+) while learning to inhibit a fear response to the safety signal (non-reinforced CS–). Results demonstrated altered levels of fear-potentiated startle (FPS) based on maternal trauma history and PTSD symptoms. The children of mothers with high levels of trauma and PTSD symptoms had higher FPS to the safety signal compared to children of mothers with moderate trauma exposure, reflecting an impairment in the ability to discriminate between danger and safety signaling appropriately. This may reflect maladaptive physiological responses to non-threatening situations. Together, the physiological and neurobiological embedding of parental trauma on child developmental outcomes emphasize the need for targeted interventions to mitigate the effects of trauma across generations.

## 4. Interventions

Intervention techniques aimed at mitigating the effects of parental trauma on children focus on both individual and family-based approaches. Although numerous interventions exist, our review aims to briefly highlight those that may mitigate the neurobiological effects of intergenerational risk and promote resilience through targeted enhancement of the parent–child relationship, family-level processes or the parent specifically. One prominent evidence-based intervention technique is the Attachment and Biobehavioral Catch-up (ABC) developed by Mary Dozier and colleagues [78,79]. This 10-week, in-home, early parenting intervention is designed for low-income families experiencing high levels of adversity, with the goal of enhancing parental sensitivity and parent–child attachment. ABC was initially developed specifically for children in foster care but has been adapted to broader family environments. Findings across multiple empirical studies demonstrate efficacy in improving sensitivity [80] and reducing parental withdrawal [81]. Additionally, these findings extend to offspring outcomes, with positive associations shown between ABC and enhanced cognitive flexibility in toddlers [82]. Results from a randomized control trial conducted by Bernard et al. [83] demonstrated differences in cortisol regulation patterns in neglected children, between the ages of 5 and 34.2 months, who received the ABC compared to controls. Children randomly assigned to the ABC intervention exhibited higher morning cortisol levels and a greater reduction in bedtime levels, suggesting less blunted cortisol and overall enhanced cortisol regulation [83] when exposed to adverse environments. This finding closely mirrors cortisol patterns often seen in children unexposed to similar adversities. Valadez at al. [84] extended these findings using a longitudinal study in which parents of high-risk infants were randomly assigned to an ABC or control intervention group. As part of an eight-year follow-up, children, ranging in age from 8 to 12 years underwent an fMRI scan while viewing pictures of their mother and a stranger. The children of parents assigned to the ABC intervention showed enhanced activity in brain regions critical for cognitive development, compared to the children of parents assigned to the control intervention. Interestingly, these findings suggest that targeting parental sensitivity has significant effects on their child’s cortisol regulation and neural activity, even in the absence of the direct intervention with the child [84].

Dyadic interventions, such as Child–Parent Psychotherapy (CPP), directly targeting the parent–child relationship aim to enhance attachment security and reduce the child’s stress response by creating a safer and more emotionally supportive environment. Sullivan et al. [85] found that participation in a CPP program led to lower epigenetic aging, which is a biomarker that characterizes higher allostatic load and accelerated aging in traumatized populations. Family-centered interventions also emphasize improving communication, emotion attunement, and co-regulation within the family system. Mindfulness-based interventions, such as parenting mindfully have shown promise in reducing parental stress and increasing empathy, especially in highly stressed adolescent parents [86].

Individual trauma-focused therapy for parents can also have beneficial impacts on child outcomes. Currently, the top three most recommended individual therapeutic interventions for PTSD are Prolonged Exposure (PE), Cognitive Processing Therapy (CPT), and Cognitive Behavioral Therapy [87]. While studies have not specifically examined the impact of such individual therapy on parenting behaviors, therapeutic engagement may increase parental responsiveness, warmth, and support, in turn buffering the adverse consequences for their children. Integrating multimodal approaches, such as combining individual trauma therapy with evidence-based techniques (e.g., ABC) can offer benefits across generations. These interventions underscore the critical role of addressing both parental trauma and its ripple effects on children, fostering long-term post-traumatic growth and resilience. Improvements in parents’ emotion regulation and stress management can foster more consistent and nurturing caregiving, thereby promoting positive outcomes in children [7].

## 5. Conclusions and Future Directions

This review underscores the critical role of parenting as a mechanism through which caregiver trauma may influence children’s developmental trajectories, both psychologically and neurobiologically. Drawing on the intergenerational transmission of trauma (ITT) framework [3], we synthesize evidence illustrating how trauma-related disruptions in caregiving, such as altered emotion regulation, attachment difficulties, and maladaptive parenting styles contribute to heightened risk for psychopathology and altered brain development in children. The literature also identifies parenting behaviors, particularly warmth and responsiveness as potential protective factors that may buffer these adverse outcomes. By integrating findings across behavioral, physiological, and neuroimaging studies, one of the key strengths of this review lies in offering a more holistic understanding of how parental trauma influences child development and identifies promising directions for trauma-informed intervention. The application of the ITT framework to psychological and neurobiological outcomes across infancy, childhood, and adolescence that are associated with parental trauma exposure enables a focused exploration of relational mechanisms while highlighting opportunities for intervention. Additionally, by incorporating evidence from both empirical studies and applied interventions, this review bridges the gap between theory and practice.

However, several limitations should be acknowledged. First, as a conceptual review, this paper does not employ systematic review protocols, which may introduce selection bias. Second, many of the studies reviewed provide valuable correlational evidence with limited longitudinal or causal research to fully establish the directionality of effects. While substantial evidence links caregiver trauma exposure to changes in parenting behavior and subsequent child neurodevelopmental outcomes, more research is needed to disentangle these pathways, examine moderating factors such as child temperament and social support, and develop trauma-informed parenting. Even further, there is a dearth of studies involving fathers and non-parental caregivers (i.e., grandparents, aunts, etc.), despite 5–6% of fathers being the sole caregiver [88] and approximately 3% of children currently living with non-parental caregivers [89]. The inclusion of father and non-parental caregivers in research will not only expand the ITT framework but also allow for the investigation of gendered expressions of parenting and trauma exposure, the dynamics of multiple caregiving influences, and advancement toward more culturally inclusive and comprehensive intervention techniques.

Future research should continue to explore the complex interactions between biological, psychological, and environmental factors of both parent and child focusing on the understudied impacts on the neurodevelopment of offspring, sex-specific effects, and the nuanced roles of parenting as either a moderator or mediator in shaping childhood neurodevelopmental outcomes within the context of parental trauma history. Furthermore, additional research is necessary to explore how these associations unfold over varying timeframes providing greater clarity on the immediate, intermediate, and long-term effects of parental trauma on child development and well-being. Lastly, the field would benefit from further investigations into preventative and intervention techniques that are culturally informed and how they impact child neurodevelopment.
